# Individual Diet Modeling Shows How to Balance the Diet of French Adults with or without Excessive Free Sugar Intakes

**DOI:** 10.3390/nu9020162

**Published:** 2017-02-20

**Authors:** Anne Lluch, Matthieu Maillot, Rozenn Gazan, Florent Vieux, Fabien Delaere, Sarah Vaudaine, Nicole Darmon

**Affiliations:** 1 Danone Nutricia Research, Centre Daniel Carasso, RD128, 91767 Palaiseau, France; fabien.delaere@danone.com (F.D.); sarah.vaudaine@danone.com (S.V.); 2 MS-Nutrition, 13005 Marseille, France; matthieu.maillot@ms-nutrition.com (M.M.); rozenn.gazan@ms-nutrition.com (R.G.); florent.vieux@ms-nutrition.com (F.V.); 3 Nutrition, Obesity and Risk of Thrombosis, Aix-Marseille Université, Institut National de la Recherche Agronomique (INRA) 1260, 13005 Marseille, France; nicole.darmon@univ-amu.fr; 4 Markets, Organizations, Institutions and Stakeholders Strategies, Institut National de la Recherche Agronomique (INRA) 1110, 34000 Montpellier, France

**Keywords:** sugars, linear programming, nutrient recommendations, dietary habits, snacking, France, INCA2

## Abstract

Dietary changes needed to achieve nutritional adequacy for 33 nutrients were determined for 1719 adults from a representative French national dietary survey. For each individual, an iso-energy nutritionally adequate diet was generated using diet modeling, staying as close as possible to the observed diet. The French food composition table was completed with free sugar (FS) content. Results were analyzed separately for individuals with FS intakes in their observed diets ≤10% or >10% of their energy intake (named below FS-ACCEPTABLE and FS-EXCESS, respectively). The FS-EXCESS group represented 41% of the total population (average energy intake of 14.2% from FS). Compared with FS-ACCEPTABLE individuals, FS-EXCESS individuals had diets of lower nutritional quality and consumed more energy (2192 vs. 2123 kcal/day), particularly during snacking occasions (258 vs. 131 kcal/day) (all *p*-values < 0.01). In order to meet nutritional targets, for both FS-ACCEPTABLE and FS-EXCESS individuals, the main dietary changes in optimized diets were significant increases in fresh fruits, starchy foods, water, hot beverages and plain yogurts; and significant decreases in mixed dishes/sandwiches, meat/eggs/fish and cheese. For FS-EXCESS individuals only, the optimization process significantly increased vegetables and significantly decreased sugar-sweetened beverages, sweet products and fruit juices. The diets of French adults with excessive intakes of FS are of lower nutritional quality, but can be optimized via specific dietary changes.

## 1. Introduction

In the current context of rising prevalence of non-communicable diseases, sugar intake is increasingly singled out as a public health issue because of its implication in dental caries [1] and weight gain [2], and potentially type 2 diabetes [3,4] and cardiovascular diseases [5,6,7]. Additionally, higher intakes of added sugars seem to be associated with poorer diet quality and lower micronutrient intakes [8]. Evidence ranges depending on health issues and sugar forms. However, the World Health Organization (WHO) recently focused on the prevention and control of unhealthy weight gain and dental caries, making recommendations for the intake of free sugars in adults and children [9].

For the WHO, the term “sugars” refers to all mono- and disaccharides, and “added sugars” include mono- and disaccharides added to food and beverages by the manufacturer, cook or consumer, and sugars naturally present in honey and syrups, while “free sugars” comprise added sugars plus sugars from fruit juices and concentrates [10].

The WHO recommends reducing the intake of free sugars to less than 10% of energy intake for both adults and children [9]. Today this is the most widely recognized recommendation, though the WHO concurrently makes a “conditional recommendation” of less than 5% of energy intake from free sugars, a threshold adopted by the Scientific Advisory Committee on Nutrition in the UK [11]. More recently, the 2015–2020 Dietary Guidelines for Americans (DGA) recommended limiting energy intakes from added sugars to a maximum of 10% [12]. In Europe, the European Food Safety Agency (EFSA) Panel on “Dietetic Products, Nutrition, and Allergies” declared in 2010 that “there are insufficient data to set an upper limit for (added) sugar intake” [13]. Similarly, in France, no recommendation has been set yet for free sugars.

Worldwide intakes of sugars vary widely by country [13,14,15,16] and subject characteristics, such as age [17] and eating patterns, including snacking habits [18]. Additionally, levels of information on sugar intakes (total, added, and free sugars) differ widely among food surveys, with little or no data on free sugars.

In this study, we were able for the first time to characterize the diet of French adults with excessive free sugar intakes, in comparison with those with acceptable free sugar intakes. We then determined the minimum dietary changes needed to achieve adequacy for all nutrients—including 10% maximum energy from free sugars—using diet modeling in individuals with and without excessive intakes of free sugars.

## 2. Materials and Methods

### 2.1. Dietary Survey and Population Sample

Data from the French national cross-sectional food consumption survey, named INCA2 (étude Individuelle Nationale des Consommations Alimentaires, 2006–2007) were used in this analysis. This cross-sectional survey, performed on nationally representative samples of children (3–17 years) and adults (18–79 years), using a multi-stage cluster sampling technique, has been described elsewhere [19,20]. To ensure national representativeness, each individual was assigned a weighting factor for unequal sampling probabilities and for differential non-responses. In terms of ethics of human subject participation, this survey was approved by the CNIL, the French authority of data protection (CNIL: “Commission Nationale Informatique et Libertés” No. 2003X727AU) and the CNIS, the French national council for statistical information (CNIS: “Conseil National de l’Information Statistique”). Verbal informed consent was obtained from all participants and formally recorded. The present study focuses on the adult population, aged between 20 and 75 years (*n* = 2486). Under-reporting individuals (i.e., those who have under-reported their food intake, voluntarily or not), were identified using the Goldberg method, based on the deviation between total energy reported and estimation of energy requirement (based on age, gender, weight, height, physical activity) [21] and excluded from the analysis (26.9% of the total adult sample). Additionally, only respondents who participated in the study for all seven days were retained, which left a final sample of 1726 individuals (Figure S1).

### 2.2. Demographic, Socio-Economic, Behavioral and Anthropometric Variables

Age, gender, socio-professional status, household type and income, current smoking status, sedentary behavior, frequency of snacking occasions and interest in diet were collected using self-reported and face-to-face questionnaires. Socio-professional status was classified as “active”, “unemployed”, “student”, “retired” or “homemaker”. The household type was described as: “in couple with at least one child”, “in couple with no child”, “single with at least one child” or “single with no child”. Income per consumption unit (ICU) was calculated as self-reported household total net income divided by the number of consumption units in the household, calculated using the scale from INSEE, the French national institute of statistics and economic studies (INSEE: “Institut National de la Statistique et des Etudes Economiques”) [22]. Smoking status was divided into “smoker” and “non-smoker”. Frequency of eating between the 3 main meals (breakfast, lunch, and dinner), as declared was divided in five frequencies (“more than four per day”, “2–3 times per day”, “one time per day”, “less than one time per day”, or “never”). Three levels of physical activity (“low”, “moderate”, or “high”) were determined according to the short version of the International Physical Activity Questionnaire (IPAQ) [23]. A variable assessing time spent looking at a screen was used as a proxy for sedentariness. This variable was calculated as the sum of the time declared spent in front of the television and computer (including at work), during the week preceding the diet record (minutes (min) per day) [20].

Interest in diet was classified into “a lot”, “little”, “not really” and “not at all”. Trained interviewers measured individual weight and height to calculate body mass index (BMI), divided into four classes (underweight, normal weight, overweight, obesity), according to the WHO definition [24].

### 2.3. Dietary Assessment

In a seven-day dietary diary, individuals recorded each food and each beverage consumed at home or outside home, split into six moments of consumption: three main meals (breakfast, lunch, and dinner) and three snacking occasions defined as food or beverage consumption between meals (morning, afternoon or evening). During the first face-to-face interview, the diary and a self-administered questionnaire were delivered at home by a trained and certified investigator, who explained to the subjects how to complete them. Just after the survey week, the investigator came back and checked the accuracy of the information reported in both documents [19]. Participants were told to complete the diary during the day in as close as real time as possible, in a pen and paper format. Portion sizes were estimated using a photographic booklet [25] or expressed by weight or household measures (spoon). All foods declared as consumed by the individual during the survey (*n* = 1314 foods and non-alcoholic beverages, including water) were placed in nine food categories and 30 sub-categories. In addition, to differentiate intrinsic sugar from free sugars, “fruits”, “milk” and “yogurts” sub-categories were split into “fresh fruits” and “processed fruits”; ”plain milk” and “sweet milk”; “plain yogurts” and “sweet yogurts”. The “yogurts” sub-category included yogurts, fermented milks and associated French specialties (“fromage blanc” and “petit-suisses”). Alcoholic beverages were excluded from food analyses because they are not considered as food sources of essential nutrients in dietary recommendations, and therefore could not be optimized.

### 2.4. Food Composition Database and Free Sugars

The French food composition database [26] was used to estimate the energy and nutrient content of diets. We completed the national food composition table with an additional variable giving the free sugar content of foods. We used the WHO definition [10] which defines free sugars as all monosaccharides and disaccharides added to foods and beverages by the manufacturer, cook or consumer, and sugars naturally present in honey, syrups, fruit juices and fruit juice concentrates. Based on the systematic method to estimate added sugar content [27], the amount of equivalent sugars in all assimilated sugar ingredients was estimated using converting factors (e.g., equivalent sugars accounted for 100% in white sugar and only 80% in honey). Finally, the amount of free sugars for 100 g was estimated using the weight (in the recipe) of assimilated sugar ingredients and their corresponding amounts of sucrose.

In foods from the French food composition table [26], free sugars equal total sugars for 98 foods: honey, 2 syrups and 95 beverages including water. For 627 foods, the amount of free sugars was estimated using average recipes developed by ANSES, the French agency for food, environmental and occupational health and safety (ANSES: “Agence Nationale de SEcurité Sanitaire de l’alimentation, de l’environnement et du travail”) and by nutritional expertise. For the remaining 589 foods considered with no recipe (mainly mono-ingredient foods such as vegetables, non-processed fruits, meats, eggs, fish, etc.), the amount of free sugars was estimated by nutritional expertise, and was nil for 538 of them.

### 2.5. Diet Quality Indicators

Solid energy density (SED), food variety, mean adequacy ratio (MAR), mean excess ratio (MER), and a diet quality index based on the Probability of Adequate Nutrient intake (PANDiet) were used as indicators of diet quality, and were estimated for each individual observed diet. SED (kcal/100 g) was calculated based on items typically consumed as foods, including soups, but excluded drinking water and items typically consumed as beverages, such as milk, juices and soft drinks [28]. SED was calculated by dividing energy provided by solid foods by their weight. A high SED is associated with low diet quality [29]. Food variety was assessed by the number of different foods declared as consumed by each individual during the 7 days food record [30,31]. As originally proposed, the MAR was used as an indicator of good nutritional quality, and was calculated for each individual observed diet as mean percentages (capped at 100%) of recommended intakes [32] over a week for a list of nutrients. In the present study, it was calculated for 23 key nutrients [33]. The MER, an indicator of poor nutritional quality, was calculated as the mean percentages (minus 100%) of maximum recommended values over a week for sodium, saturated fatty acids and added sugars [33,34]. The updated version of the PANDiet index, integrating free sugars, was also used to estimate the overall nutritional quality of individual diets [35]. It summarizes in a single score the probability of having adequate intakes for 25 positive and negative nutrients. The score ranges from 0 to 100; the higher the score, the better the nutrient adequacy of the diet.

### 2.6. Diet Modeling

The present modeling approach was based on the previously described Individual Diet models (ID models) [36]. However, to improve its relevance, some changes were made to the original ID models and are described in the Supplementary Materials—Methods. Briefly, the present modeling approach was used to design, for each individual in the dietary survey, a diet at the same energy level which met a set of 33 nutritional recommendations (including 10% maximum energy from free sugars if the intake was greater than 10% or a “no increase” constraint when energy from free sugars was lower than or equal to 10%), while departing the least from the observed diet. To design a diet as similar as possible to the corresponding observed one, the model was parameterized to: (i) preferentially choose repertoire foods (i.e., foods declared as consumed by the individual); (ii) minimize the reduction of the repertoire foods; and (iii) control the introduction of non-repertoire foods (i.e., foods declared as consumed at least once in the survey, but not by this individual). The constraints to be met were a set of nutritional constraints based on dietary reference intakes, a set of acceptability constraints (maximum amounts of foods and food groups) and a set of other constraints, in particular total diet weight and total diet cost (Table S1). “Energy-free” drinks (i.e., drinks containing less than 4 kcal/100 mL) were excluded from the calculation of total diet weight to avoid competition between energy-free drinks and nutrient-dense foods with low energy content.

### 2.7. Identification of the Most Binding Nutrients

It is possible to identify the constraints the most difficult to fulfill by calculating, for each constraint, a factor named dual value. A null dual value indicates that the constraint is inactive: it has no impact on the optimized solution. In contrast, a non-null dual value means that the constraint is binding or active: it is influencing the result of the optimization process. To identify the most binding constraint, nutritional constraints were ranked in decreasing order according to their percentage of non-null dual values, estimated on the 1719 individuals.

### 2.8. Statistical Analyses

Of the 1726 adults, the diet optimization was unfeasible for 7 (i.e., no modeled diet able to simultaneously meet all the constraints could be mathematically designed with the list of food variables available for diet modeling). A final sample of 1719 adults was therefore taken for the statistical analysis. Two groups of individuals were defined, depending on the energy contribution from free sugars in their observed diets. Based on the WHO recommendation [9], individuals with a contribution greater than 10% were assigned to the “FS-EXCESS” group (excessive free sugar intakes), and those who had a contribution lower or equal to 10% were assigned to the “FS-ACCEPTABLE” group (acceptable free sugar intakes).

Individual characteristics were described and compared between FS-ACCEPTABLE and FS-EXCESS groups using a chi-squared test for categorical variables and general linear model (GLM) for continuous variables, with and without adjustment for gender and age.

Mean observed nutritional intakes, diet quality indicators, and intakes from food categories and sub-categories (as well as from fresh and processed fruits, plain and sweet milk and plain and sweet yogurts) were described for the whole sample and the two groups. Comparisons of observed food intake, nutritional intake and diet quality indicators between FS-EXCESS and FS-ACCEPTABLE individuals were made using GLM. Observed energy and free sugar intakes from main meals and from snacking occasions were also described and compared between the FS-ACCEPTABLE and FS-EXCESS groups with GLM.

GLM were used to compare the characteristics of observed and optimized diets in the two groups and to compare the variation in grams between optimized and observed diets among FS-EXCESS and FS-ACCEPTABLE individuals. To study changes in sugar balance after diet modeling, the variation in total, free and non-free sugars between observed and optimized diets, from main food categories and sub-categories were calculated and compared using GLM.

Observed energy intake, age and gender were used as a first set of adjustment variables. In a second set of adjustment variables, the current smoking status, BMI, socio-professional status were added to the first set, and, in a third set of adjustment variables, the composition of the family and sitting time were added to the second set. All values were survey-weighted and all analyses accounted for the complex INCA2 sampling frame design [19]. The Operational Research and the STAT packages of SAS version 9.4 (SAS Institute, Cary, NC, USA) were used to run linear programming models and perform statistical analysis, respectively. An alpha level of 1% was used for all statistical tests.

## 3. Results

### 3.1. Sample Characteristics

In this representative sample of French adults (*n* = 1693, weighted value), 41% of individuals (FS-EXCESS group, *n* = 690) had mean free sugar intakes above the 10% of energy intake level recommended by the WHO (mean intake 14.2% ± 4.2% of energy intake) and 59% (FS-ACCEPTABLE group, *n* = 1003) had acceptable intakes, i.e., below 10% of energy intake (mean intake 6.3% ± 2.5% of energy intake).

Demographic, anthropometric, socio-economic and behavioral characteristics are given in Table 1. Individuals were on average 10 years younger in the FS-EXCESS group than in the FS-ACCEPTABLE group. Individuals in the FS-EXCESS group had a lower BMI (23.6 vs. 25.2 kg/m^2^), and the percentage of overweight or obese individuals among them was lower than in the FS-ACCEPTABLE group, even after adjustment for age and gender. In the FS-EXCESS group, the percentages of single individuals and couples with children were higher than in the FS-ACCEPTABLE group, while the percentage of couples without children was lower. In addition, the percentages of professionally active people and students were higher, while the percentage of retirees was lower in the FS-EXCESS group than in the FS-ACCEPTABLE group. There were proportionately more smokers in the FS-EXCESS group; this difference between groups was no longer significant after adjustment for age and gender.

Physical activity (IPAQ) level did not significantly differ between groups. However, the FS-EXCESS individuals spent significantly more time sitting in front of computers or television (+25 min per day) than the FS-ACCEPTABLE individuals, but this difference between groups was no longer significant after adjustment for age and gender. The FS-EXCESS individuals declared that they ate more often between meals, and they were less interested in their diet; these results remained significant after adjustment for age and gender.

### 3.2. Observed Nutritional Intakes and Diet Quality Indicators

Observed nutritional intakes and diet quality indicators are detailed in Table 2. Compared with the FS-ACCEPTABLE group, individuals in the FS-EXCESS group had higher daily energy intakes (2192 vs. 2123 kcal/day), with a higher energy contribution of carbohydrates (45.4% energy vs. 40.8%) and lower energy contributions from proteins and fats (respectively 15.3% and 37.1% vs. 17.3% and 39.4%) after adjustment for age, gender and energy intake (except for energy intake, only adjusted for age and gender) or further adjustment for other sociodemographic and lifestyle parameters (see footnote to Table 2 for details). With all adjustments, energy intakes at main meals did not significantly differ between the two groups, unlike energy intakes at snacking occasions, higher in FS-EXCESS vs. FS-ACCEPTABLE groups (258 kcal/day vs. 131 kcal/day). The quantity of free sugars at each moment of consumption (meals or snacking occasion) was higher in FS-EXCESS vs. FS-ACCEPTABLE individuals. For FS-ACCEPTABLE individuals, the quantity of free sugars consumed in main meals was 4.3 times greater than at snacking occasions, while this ratio was only 2.6 for the FS-EXCESS group (data not shown). Compared with FS-ACCEPTABLE individuals, those in the FS-EXCESS group ate a more energy-dense diet (185 versus 165 kcal/100 g), and had lower nutritional quality diets, as shown by a lower PANDiet score, a lower MAR and a higher MER. For 11 out of the 23 nutrients of the MAR, capped percentages of recommended intakes were lower for FS-EXCESS group compared with FS-ACCEPTABLE group. There were no significant differences for the 12 remaining nutrients. When looking at the MER, among the three nutrients, free sugars were driving the difference between the two FS groups (Table S2).

### 3.3. Food Amounts in Observed Diets

Food amounts in the observed diets of FS-ACCEPTABLE and FS-EXCESS groups are detailed in Table 3. The amounts of fruits, vegetables, starchy foods (except ready-to-eat cereals), meat/eggs/fish, cheese, water and added fats were higher in the FS-ACCEPTABLE than in the FS-EXCESS group. By contrast, the amounts of sweet products (all sub-categories), sugar-sweetened beverages, fruit juices and sweet yogurts were higher in the FS-EXCESS than in the FS-ACCEPTABLE group. All these differences were significant after adjustment for all the variables considered, except for water (significantly different between groups after adjustment for age, gender and energy intake only).

### 3.4. Food Amounts and Weight Variations after Optimization

**Figure 1:**
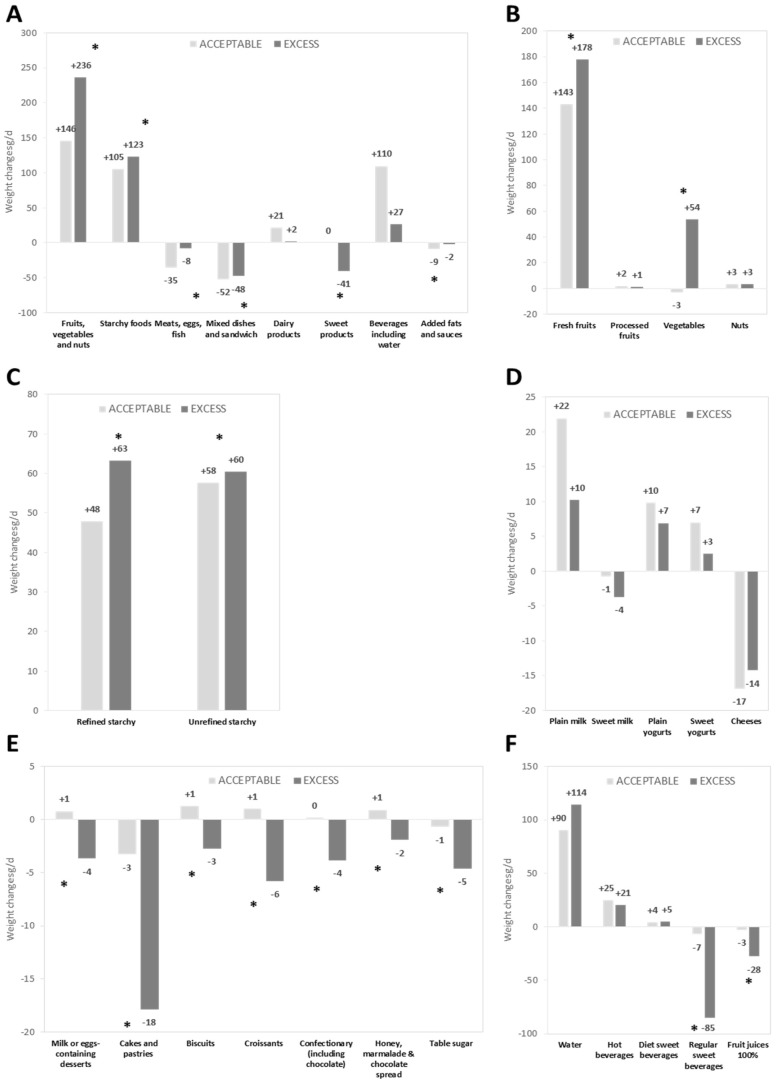
Weight changes ^1^ between observed and optimized diets (g/day) in food categories (**A**); and in food sub-categories for: fruits/vegetables/nuts (**B**); starchy foods (**C**); dairy products (**D**); sweet products (**E**); and beverages including water (**F**), in FS-ACCEPTABLE and FS-EXCESS individuals ^2^. ^1^ Italic and bold values indicate a weight change significantly different from zero adjusted for age, gender, energy intake, smoker status, BMI, socio-professional status, composition of the family and sitting time; ^2^ the * symbol means that the weight changes were significantly different between FS-ACCEPTABLE and FS-EXCESS groups, adjusted for age, gender, energy intake, smoker status, BMI, socio-professional status, composition of the family and sitting time.

Food amounts in optimized diets are given in Table 3, and food weight variations between observed and optimized diets (i.e., dietary changes induced by the optimization process) are shown in Figure 1.

At the food category level (Figure 1A), for both FS-ACCEPTABLE and FS-EXCESS individuals, the optimization process significantly increased the amount of fruits/vegetables/nuts and starchy foods, and significantly decreased the amount of meats/eggs/fish, mixed dishes/sandwiches and added fats (all *p* values < 0.001 except for added fats in FS-EXCESS, *p* = 0.012). The amount of dairy products and beverages was significantly increased for the FS-ACCEPTABLE individuals only, while sweet products were decreased for the FS-EXCESS individuals only (all *p* values < 0.001). The other changes at food category level were not significantly different from 0.

At the sub-category level, for both FS-ACCEPTABLE and FS-EXCESS individuals, fresh fruits (Figure 1B) and both refined and unrefined starchy foods (Figure 1C) were increased. The amount of vegetables was increased only for FS-EXCESS individuals (Figure 1B). Plain yogurts significantly increased and cheese decreased for both groups, whereas plain milk and sweet yogurts increased significantly only for FS-ACCEPTABLE individuals (Figure 1D). All sub-categories of sweet products were decreased for FS-EXCESS individuals (Figure 1E). For the beverage category (Figure 1F), water and hot beverage sub-categories were significantly increased for both FS-ACCEPTABLE and FS-EXCESS, whereas sugar-sweetened beverages and fruit juices were decreased only for FS-EXCESS individuals.

### 3.5. Identification of the Most Binding Nutrients

Based on dual values, the most binding constraints (in decreasing order) were those on total energy, the maximal amounts of sodium, free sugars and saturated fatty acids and the minimal amount of total carbohydrates. They presented non-null dual values for more than 75% of individuals in the total sample (data not shown).

### 3.6. Changes in Sugar Balance after Optimization

**Figure 2:**
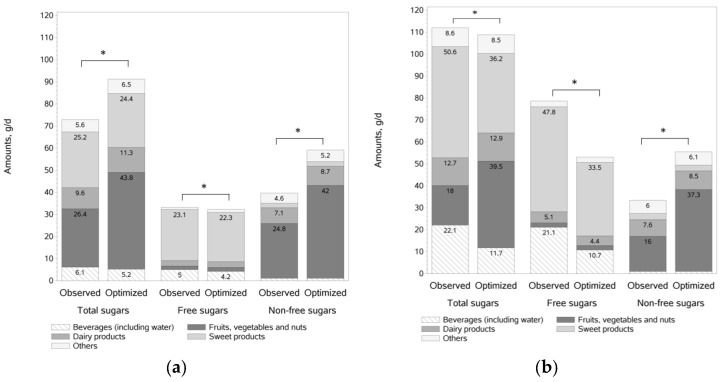
Amount of sugars (total, free, non-free) ^1^ in observed and optimized diets (g/d), from main food category contributors in: FS-ACCEPTABLE (**a**); and FS-EXCESS (**b**) individuals ^2^. ^1^ Amounts of sugars lower than 4 g not labeled; ^2^ For both FS-ACCEPTABLE and FS-EXCESS, total sugars, free sugars and non-free sugars were significantly different between observed and optimized diets after adjustment for age, gender, energy intake, smoker status, BMI, socio-professional status, composition of the family and sitting time.

The amounts of total, free and non-free sugars in the observed and optimized diets (g/day), from main food category contributors are shown in Figure 2 for FS-ACCEPTABLE and FS-EXCESS individuals.

For FS-ACCEPTABLE individuals, total sugars were significantly increased after optimization (+17.5 g/day) resulting from an increase in non-free sugars (+18.7 g/day), mainly due to an increase in fresh fruits (+16 g/day) and dairy products (+1.6 g/day) and a small decrease in free sugars (−1.2 g/day) from sweet products and beverages.

For FS-EXCESS individuals, to reach the maximum 10% energy from free sugars allowed by the model, the optimization significantly reduced free sugars (−25.5 g/day) through a decrease in sweet products (−14.3 g/day), sugar-sweetened beverages (−7.8 g/day) and fruit juices (−2.6 g/day) (Figure 2 and Table 3). Non-free sugars were significantly increased (+22.1 g/day), mainly due to an increase in fresh fruits (+19.5 g/day), in the fruits/vegetables/nuts category. All these changes led to a slight but significant decrease in total sugars (−3.4 g/day).

## 4. Discussion

In this representative sample of French adults, individuals with excessive intakes of free sugars represented 41% of the total population. Compared to the diets of individuals with acceptable free sugars intakes, their diets were found to be of lower nutritional quality, but could be optimized mostly via an increase in fresh fruits, vegetables and starchy foods, and a decrease in sweet products and sweet beverages including sugar-sweetened beverages and fruit juices.

Free sugar intakes represented 9.5% of energy intake in the French adult population. This is one of the lowest levels estimated by the WHO in European countries, and just below the WHO cut-off of 10%. Even so, individuals with free sugar intakes above this cut-off value (i.e., the FS-EXCESS group) represented 41% of the French adult population. Despite the existence of national and international recommendations on free sugars [9,11], it is currently difficult to estimate intakes of free sugars accurately, because information from nutrient composition tables is insufficient. Published studies on sugar intakes are mostly based on data on total and added sugars [13,15,16,17]. To our knowledge, only one other study recently conducted in the Dutch population [37] has also estimated free sugar intakes in a nationally representative sample. Compared with our French sample, a higher free sugar contribution (around 13.5% vs. 9.5% of energy intake) and with a lower adherence to the 10% WHO guidelines (around 30% vs. 59% respectively) was found in Dutch adults [37]. Interestingly, within the Dutch adult population, free sugar intakes decreased with age (from 16% to 11% of energy intake), in line with our findings, with FS-EXCESS individuals being 10 years younger than FS-ACCEPTABLE individuals.

Overall, in our sample, FS-EXCESS individuals had lower quality diets than FS-ACCEPTABLE individuals, as shown by a more energy-dense diet, lower MAR and PANDiet scores, and higher MER. These results are consistent with the conclusions of a recent review indicating that higher intake of added sugars is associated with poorer diet quality (in 20 out of 21 studies) and lower micronutrient intakes (in 21 out of 30 studies) [8]. Our results can be explained by food choices characterized by a lower consumption of foods of higher nutritional quality (e.g., fruits and vegetables) and a higher consumption of foods of lower nutritional quality (e.g., sweet products and sugar-sweetened beverages) [38] in FS-EXCESS individuals than in FS-ACCEPTABLE individuals.

FS-EXCESS individuals had a lower BMI than FS-ACCEPTABLE ones, despite higher energy intakes (+70 kcal/day) and greater sedentariness. A similar counterintuitive inverse relation between sugar intake and BMI was previously reviewed and discussed [39]. Selective underreporting of high sugar foods and drinks by overweight and obese people was listed as a potential explanatory factor. The present survey being based on cross-sectional data, unhealthier eating patterns and lifestyles observed in FS-EXCESS individuals, may lead over time to weight gain, the extent of which could be estimated through the use of simplified dynamic energy balance models [40].

The top three food contributors to free sugar intakes were the same for both FS-ACCEPTABLE and FS-EXCESS groups, but with different contribution levels (measured in g/day of free sugars): sweet products (23.1 g/day and 47.8 g/day for FS-ACCEPTABLE and FS-EXCESS respectively) followed by beverages (5.0 g/day and 21.1 g/day) and dairy products (2.5 g/day and 5.1 g/day). Looking at food changes needed to achieve nutrient adequacy, the optimized diets showed similarities in the FS-ACCEPTABLE and FS-EXCESS groups (increase in fresh fruits, starchy foods, water, hot beverages and plain yogurts; decrease in mixed dishes/sandwiches, meat/eggs/fish and cheese). Additional food changes were found only in FS-EXCESS individuals, and consisted in a decrease in sweet products, sugar-sweetened beverages and fruit juices. Overall, the models were aimed not only at reducing free sugars, but also at ensuring a broad set of 33 nutritional recommendations were met without changing the energy level and thereby promoting nutrient density. This explains why food sub-categories containing free sugars were not necessarily decreased after optimization. For example, sweet yogurts were significantly increased in the FS-ACCEPTABLE individuals (+70 g/week). In other words, despite their free sugar content, the ID model selected sweet yogurts as a source of nutrients to favor.

Overall, the dietary changes needed to achieve nutrient adequacy were in line with the ongoing PNNS, the French national nutrition and health program (PNNS: “Programme National Nutrition Santé”), designed to improve health by helping people eat a healthier diet [41], where particular emphasis was placed on encouraging fruit and vegetable consumption, physical activity, and the consumption of whole grains, while reducing the consumption of foods with added sugars. In the US, to meet nutrient needs within calorie limits, advice has been recently given to choose a variety of nutrient-dense foods across and within all food groups, to limit calories from added sugars and saturated fats and to reduce sodium intake [12].

The strength of the present study lies in the ability to identify and quantify the dietary changes that may help any individual meet all 33 nutrient recommendations at the same time. Diet modeling with linear programming was early described as a unique tool to help develop food based dietary guidelines and public health messages [42]. Recently, the mean UK population diet was optimized to design the new Eatwell guide [43]. In the present study, individual diet modeling was used, rather than population diet modeling, in order to take into account the variability of individual food consumption. In addition, it is only with individual diet modeling that statistical analyses can be performed, therefore providing more robust conclusions. For FS-EXCESS individuals, dietary changes would mean halving sugar-sweetened beverages and fruit juices (from one glass/day to 1/2 glass/day) and table sugar (from 2 teaspoons/day to one teaspoon/day), reducing by about 1 portion /week for cakes and pastries, whereas fresh fruits would have to be greatly increased, with the addition of two portions of 80 g per day. If some of these changes could be difficult to integrate in daily life, our results do not seem to be drastically different from advice from a Register Dietician (RD). The RD would probably focus on the few major food changes able to rebalance the diet. The objective would not be to achieve adequacy for all nutrients at the same time but to correct major mistakes related to excesses or deficiencies. Overall, where a step-by-step approach would be taken by the RD, all the changes are considered at the same time with our mathematical model. Today, both approaches could be considered as complementary.

The present study has limitations. The choice was made to exclude alcoholic beverages from the present analyses because nutrient recommendations usually apply to non-alcoholic energy intakes. Similar choices were previously made in diet modeling with linear programming studies [43,44]. However, this limitation would appear negligible, since free sugars contained in alcoholic beverages contributed to only 0.16% of total energy intakes in our population (data not shown). In addition, allowing alcoholic beverages as variables in individual diet models was recently reported as difficult to manage, because they can contribute to energy requirements and some essential nutrients (e.g., B vitamins and iron) for some individuals, without supplying any detrimental nutrients (e.g., sodium and saturated fatty acids) [44]. After optimization, the increase in total sugars seen in the FS-ACCEPTABLE group could also be considered as a limitation. However, we believe this increase would not have negative health effects since it is related to an increase in non-free sugars, mainly coming from fresh fruits. Indeed, in the absence of starch and total sugars recommendations, as fruits are more nutrient dense than starch, they are increased in large amounts by the optimization process to help meeting both micro-nutrient recommendations and the minimum carbohydrate energy contribution, leading to an increase in total sugars. Finally, the modeled diets could be questioned in terms of acceptability and feasibility. Despite its recognized interest in public health and nutrition [42], individual diet modeling with linear programming has only been used in the field of epidemiology. To improve realism, diets were optimized while staying as close as possible to current habits of each individual, such as preferentially using foods from his/her repertoire, which means that foods or drinks with lower nutritional profiles were not necessarily decreased or suppressed in the optimized diet [41]. For example, in the FS-ACCEPTABLE group, the amount of sweet products (91 g/day) did not change between observed and optimized diets. In terms of behavior change, adjusting food quantities rather than banning foods of low nutritional quality could be criticized, as some people may have difficulties keeping control over amounts consumed, highlighting the importance of considering individual psychological traits [45]. Future work could integrate complementary information on individual eating behavior characteristics, especially more refined acceptability parameters for a given individual. Generating results in portion sizes as well as integrating moments of consumption in the model would better target specific food changes in meals and snacking occasions. This would be particularly relevant for FS-EXCESS individuals studied here, as their dietary habits led to higher intakes of energy (in particular from sweet foods and drinks) specifically in snacking occasions compared with FS-ACCEPTABLE individuals. Once these model improvements have been made, these theoretical results will become more realistic, allowing some individual advice. Then, improvement of diet adequacy and metabolic parameters could be tested in a clinically relevant way.

## 5. Conclusions

In conclusion, the diet quality of French adults with excessive intakes of free sugars can be optimized by food changes that do not overly challenge their eating habits. To improve the estimation of free or added sugars and quality of food composition databases, initiatives such as the nutritional labeling of added sugars to be implemented on US food packages [46,47], are of interest to follow up. Finally, intervention studies are now needed to assess the feasibility, together with their short-term and long-term impact, of the changes in diet suggested by our study results.

## Figures and Tables

**Table 1:** Demographic, anthropometric, socio-economic and behavioral characteristics of the total sample, FS-ACCEPTABLE and FS-EXCESS groups.

	ALL	FS-ACCEPTABLE	FS-EXCESS	*p* ^1^	*p* ^2^
Individuals, *n*	1693	1003	690		
Age, year ^3^	47.0 ± 15.02	51.1 ± 14.0	41.1 ± 14.5	<0.001	-
Age, %					
20–34	25.4	15.1	40.3		
35–49	29.8	28.9	31.3		
50–64	29.0	35.0	20.3		
65–75	15.8	21.0	8.2		
Gender, %				0.087	-
Male	47.6	49.4	45.0		
Female	52.4	50.6	55.0		
BMI, kg/m^2 3,4^	24.5 ± 4.3	25.2 ± 4.3	23.6 ± 4.0	<0.001	0.001
BMI, %				<0.001	0.001
<18.5 kg/m^2^	4.2	3.0	6.0		
18.5 to <25 kg/m^2^	55.7	49.2	65.1		
25 to <30 kg/m^2^	30.6	35.4	23.7		
>30 kg/m^2^	9.5	12.4	5.3		
Household composition, %					
Couple with at least one child	31.7	28.2	36.8	<0.001	
Couple with no child	42.3	49.3	32.0		
Single with at least one child	5.4	4.2	7.0		
Single with no child	20.6	18.3	23.9		
Missing information	0.1	.	0.2		
Socio-professional status, %				<0.001	
Active	56.2	52.6	61.5		
Unemployed	4.2	3.4	5.3		
Student	4.9	1.6	9.6		
Retired	25.9	34.3	13.8		
Homemaker	8.8	8.1	9.8		
ICU, euros/month ^3^	1328 ± 837	1359 ± 830	1285 ± 847	0.069	
Current smoking status, %				<0.001	0.0216
Smoker	27.9	23.7	34.0		
Non-smoker	70.3	74.8	63.7		
Missing	1.8	1.5	2.3		
IPAQ, %				0.156	0.239
Low	22.6	20.9	25.1		
Moderate	30.5	31.2	29.6		
High	45.7	46.8	44.0		
Missing information	1.2	1.1	1.3		
Screen for leisure time, minutes/day ^3,4^	205 ± 138	195 ± 128	221 ± 151	0.002	0.020
In front of computer	60 ± 97	52 ± 94	73 ± 101	<0.001	0.249
In front of TV	145 ± 98	143 ± 89	148 ± 110	0.442	0.040
Frequency of eating between meals, as declared %				<0.001	<0.001
≥4 times/day	2.4	1.3	4.0		
2 to 3 times/day	15.1	11.8	19.8		
1/day	31.5	28.1	36.3		
>0 and <1/day	25.3	26.6	23.4		
Never	23.4	29.6	14.4		
Missing/invalid answers	2.4	2.6	2.1		
Interest in diet, %				0.001	0.010
A lot	32.8	36.4	27.7		
Little	44.7	44.6	45.0		
Not really	16.3	13.3	20.7		
Not at all	4.9	4.5	5.3		
Missing/invalid answers	1.3	1.2	1.3		

Abbreviations: BMI, body mass index; ICU, income per consumption unit; IPAQ, International Physical Activity Questionnaire. ^1^ *p* value provided by chi-squared test for categorical variables and GLM for continuous variables; ^2^ Gender-age adjusted *p* values provided by logistic regression for categorical variables and GLM for continuous variable; ^3^ Results are Mean ± SD; ^4^ One missing information items for BMI and seven missing information items for screen for leisure time variable.

**Table 2:** Observed nutritional intakes and diet quality indicators for the total sample and for FS-ACCEPTABLE and FS-EXCESS groups (mean ± SD).

	ALL	FS-ACCEPTABLE	FS-EXCESS	*p* ^1^	*p ^2^*	*p* ^3^
Individuals, *n*	1693	1003	690			
	Mean ± SD					
Energy intake (kcal/day) ^4^	2151 ± 536	2123 ± 539	2192 ± 529	0.016	0.007	0.008
from main meals (kcal/day)	1969 ± 501	1992 ± 509	1935 ± 487	0.158	0.316	0.359
from snacking occasions (kcal/day)	183 ± 194	131 ± 154	258 ± 220	<0.001	<0.001	<0.001
Proteins, % of energy	16.5 ± 2.7	17.3 ± 2.7	15.3 ± 2.2	<0.001	<0.001	<0.001
Fats, % of energy	38.5 ± 5.7	39.4 ± 6.0	37.1 ± 4.8	<0.001	<0.001	<0.001
Carbohydrates, % of energy	42.7 ± 6.1	40.8 ± 6.2	45.4 ± 5.0	<0.001	<0.001	<0.001
Free sugars, % of energy	9.5 ± 5.1	6.3 ± 2.5	14.2 ± 4.2	<0.001	<0.001	<0.001
Starch, g/day	141.1 ± 51.2	143.5 ± 55.7	137.7 ± 43.6	<0.001	<0.001	<0.001
Total sugars, g/day	90.2 ± 37.3	75.1 ± 29.2	112.1 ± 37.1	<0.001	<0.001	<0.001
Free sugars g/day	51.9 ± 33.1	33.5 ± 16.6	78.7 ± 33.1	<0.001	<0.001	<0.001
from main meals (g/day)	39.4 ± 24.5	27.2 ± 14.4	57.0 ± 25.5	<0.001	<0.001	<0.001
from snacking occasions (g/day)	12.6 ± 16.3	6.3 ± 7.2	21.7 ± 20.9	<0.001	<0.001	<0.001
Non-free sugars, g/day	38.3 ± 19.6	41.6 ± 21.1	33.4 ± 16.0	<0.001	<0.001	<0.001
Alcohol, g/day	0.22 ± 0.74	0.18 ± 0.63	0.27 ± 0.87	0.086	0.052	0.050
Solid energy density, kcal/100 g	173.4 ± 32.7	165.2 ± 14.9	185.3 ± 31.6	<0.001	<0.001	<0.001
Variety, number of foods/week	58.4 ± 14.9	57.4 ± 7.6	60.0 ± 14.7	0.017	0.006	0.014
PANDiet	62.7 ± 7.5	64.3 ± 12.8	60.4 ± 6.6	<0.001	<0.001	<0.001
MAR, %	83.8 ± 9.0	84.8 ± 23.9	82.4 ± 9.3	<0.001	<0.001	<0.001
MER, %	32.2 ± 30.0	25.1 ± 14.9	42.6 ± 34.6	<0.001	<0.001	<0.001

Abbreviations: PANDiet, probability of adequate nutrient intake; MAR, mean adequacy ratio; MER, mean excess ratio. ^1^ GLM with survey design adjusted for age, gender and energy intake (except for energy intake, adjusted for age and gender only); ^2^ GLM with survey design adjusted for age, gender, energy intake, smoking status, BMI and socio-professional status (except for energy intake, adjusted for age and gender only); ^3^ GLM with survey design adjusted for age, gender, energy intake, smoking status, BMI, socio-professional status, composition of the family and sitting time (except for energy intake, adjusted for age and gender only); ^4^ 1 kcal =4.184 kJ.

**Table 3:** Amounts of food categories and sub-categories in observed and optimized diets for the total sample and for FS-ACCEPTABLE and FS-EXCESS groups (g/day).

		Observed Diets			Optimized Diets				FS-Acceptable	FS-Excess
	ALL	FS-ACCEPTABLE	FS-EXCESS	*p* ^1^	ALL	FS-ACCEPTABLE	FS-EXCESS	*p* ^1^	*p* ^2^	*p* ^2^
Individuals, *n*	1693	1003	690		1693	1003	690			
	**Mean ± SD**				**Mean ± SD**					
Fruits/vegetables/nuts	379.7 ± 236.7	438.0 ± 248.1	294.8 ± 189.8	a,b,c	562.3 ± 177.2	583.8 ± 184.3	531.1 ± 161.4	a,b,c	<0.001	<0.001
Fruits	163.1 ± 148.8	190.8 ± 162.9	122.9 ± 114.1	a,b,c	322.3 ± 138.9	336.1 ± 144.9	302.2 ± 127.1	a,b,c	<0.001	<0.001
Fresh	149.6 ± 144.4	178.5 ± 159.1	107.4 ± 106.6	a,b,c	307.0 ± 137.2	321.8 ± 143.5	285.4 ± 124.5	a,b,c	<0.001	<0.001
Processed	13.6 ± 31.7	12.3 ± 31.1	15.5 ± 32.4	a,b,c	15.3 ± 31.9	14.3 ± 32.8	16.8 ± 30.4		<0.001	0.063
Vegetables	214.5 ± 142.3	245.2 ± 149.6	169.8 ± 117.4	a,b,c	234.6 ± 99.4	242.3 ± 102.8	223.4 ± 93.2		0.593	<0.001
Nuts	2.1 ± 5.8	2.0 ± 5.7	2.1 ± 6.0		5.4 ± 9.2	5.4 ± 9.3	5.5 ± 9.1		<0.001	<0.001
Starchy foods	254.2 ± 118.9	270.0 ± 127.1	231.2 ± 101.6	a,b,c	366.7 ± 109.4	375.4 ± 112.2	354.1 ± 104.1	a,b,c	<0.001	<0.001
Refined	168.8 ± 96.4	181.2 ± 102.5	150.7 ± 83.5	a,b,c	222.9 ± 92.2	229.1 ± 94.2	214.0 ± 88.4	a,b,c	<0.001	<0.001
Unrefined	80.5 ± 59.4	85.1 ± 64.6	73.9 ± 50.1	a,b,c	139.3 ± 56.6	142.7 ± 58.1	134.3 ± 53.8	a,b,c	<0.001	<0.001
Ready-to-eat cereals	4.9 ± 16.3	3.6 ± 13.4	6.7 ± 19.6		4.5 ± 14.2	3.6 ± 13.1	5.9 ± 15.5		0.881	0.033
Meats/eggs/fish	166.9 ± 70.2	179.9 ± 71.7	147.9 ± 63.4	a,b,c	142.8 ± 43.2	144.5 ± 42.2	140.2 ± 44.7		<0.001	0.001
Mixed dishes and sandwiches	120.9 ± 91.4	117.4 ± 93.7	125.9 ± 87.9		70.4 ± 53.8	65.1 ± 51.9	78.1 ± 55.6		<0.001	<0.001
Dairy products	209.4 ± 173.7	201.5 ± 174.4	221.0 ± 172.1		222.7 ± 146.0	222.7 ± 142.1	222.7 ± 151.6		<0.001	0.754
Milk	94.4 ± 151.6	85.5 ± 153.1	107.5 ± 148.4		109.7 ± 138.0	106.7 ± 133.2	114.1 ± 144.7		<0.001	0.183
Plain milk	86.8 ± 145.8	80.9 ± 151.6	95.5 ± 136.5		104.0 ± 134.3	102.9 ± 132.9	105.7 ± 136.4		<0.001	0.034
Sweet milk	7.6 ± 38.2	4.6 ± 28.1	12.0 ± 49.0		5.7 ± 29.3	3.8 ± 23.7	8.3 ± 35.8		0.202	0.004
Yogurts	80.9 ± 81.3	78.9 ± 79.9	83.7 ± 83.1		94.7 ± 83.6	95.8 ± 83.3	93.2 ± 84.0		<0.001	0.000
Plain yogurts	39.5 ± 60.5	44.8 ± 67.0	31.7 ± 48.6		48.1 ± 66.1	54.7 ± 71.6	38.7 ± 55.9		<0.001	<0.001
Sweet yogurts	41.4 ± 59.5	34.1 ± 53.4	52.0 ± 66.0	a,b,c	46.6 ± 61.4	41.1 ± 58.4	54.5 ± 64.8		<0.001	0.227
Cheese	34.1 ± 28.8	37.1 ± 29.8	29.8 ± 26.9	a,b,c	18.3 ± 14.0	20.2 ± 14.5	15.5 ± 12.9		<0.001	<0.001
Sweet products	119.9 ± 76.0	91.1 ± 58.7	161.7 ± 79.2	a,b,c	103.4 ± 65.5	91.3 ± 62.5	121.2 ± 65.7	a,b,c	0.907	<0.001
Milk or eggs-containing desserts	18.4 ± 31.0	12.3 ± 21.7	27.3 ± 39.3	a,b,c	17.4 ± 29.4	13.1 ± 23.9	23.6 ± 35.2	a,b,c	0.082	<0.001
Cakes and pastries	48.3 ± 44.7	41.1 ± 38.5	58.9 ± 50.6	a,b,c	39.1 ± 37.1	37.7 ± 36.3	41.0 ± 38.1		0.006	<0.001
Biscuits	8.2 ± 18.4	4.6 ± 10.8	13.6 ± 24.9	a,b,c	7.9 ± 15.3	5.8 ± 12.5	10.8 ± 18.1	a,b,c	<0.001	0.001
Croissants	17.5 ± 26.0	14.2 ± 23.6	22.1 ± 28.5	a,b,c	15.7 ± 24.3	15.3 ± 24.5	16.4 ± 24.0		0.267	<0.001
Confectionery (incl. chocolate)	5.8 ± 14.1	3.1 ± 6.4	9.9 ± 20.0	a,b,c	4.4 ± 7.9	3.2 ± 6.2	6.0 ± 9.6	a,b,c	0.229	<0.001
Honey, marmalade and chocolate spread	13.8 ± 19.8	10.4 ± 15.8	18.6 ± 23.8	a,b,c	13.5 ± 17.5	11.3 ± 16.3	16.7 ± 18.7	a,b,c	0.009	0.003
Table sugar	7.8 ± 10.2	5.5 ± 7.6	11.3 ± 12.4	a,b,c	5.5 ± 6.9	4.8 ± 6.7	6.7 ± 7.0	a,b,c	<0.001	<0.001
Beverages including water	1322.6 ± 630.3	1288.5 ± 655.7	1372.1 ± 588.3		1398.4 ± 403.0	1398.1 ± 421.3	1398.8 ± 374.9		<0.001	0.259
Water	798.6 ± 569.3	821.1 ± 596.0	765.7 ± 526.7	a	898.4 ± 412.1	911.2 ± 426.8	879.8 ± 389.3		<0.001	<0.001
Hot beverages	389.4 ± 335.3	403.7 ± 332.1	368.4 ± 339.0		412.4 ± 334.3	428.4 ± 325.0	389.1 ± 346.2		<0.001	<0.001
Diet beverages	12.9 ± 62.2	10.2 ± 58.3	16.9 ± 67.3		17.6 ± 72.2	14.6 ± 69.2	21.9 ± 76.3		0.018	0.009
Sugar-sweetened beverages	62.4 ± 175.4	17.0 ± 46.4	128.3 ± 255.5	a,b,c	23.5 ± 60.8	10.1 ± 31.1	43.1 ± 83.9	a,b,c	<0.001	<0.001
Fruit juices 100%	59.4 ± 89.9	36.4 ± 62.2	92.8 ± 111.4	a,b,c	46.5 ± 67.2	33.7 ± 55.0	65.0 ± 78.4	a,b,c	0.012	<0.001
Added fats and sauces	45.5 ± 23.4	48.1 ± 23.3	41.8 ± 23.1	a,b,c	39.4 ± 18.4	39.2 ± 19.0	39.8 ± 17.6		<0.001	0.012
Foods based on soya	3.5 ± 25.4	4.0 ± 25.9	2.9 ± 24.7		3.8 ± 25.5	4.0 ± 25.4	3.5 ± 25.6		0.907	0.168

Abbreviations: FS, free sugars; incl. chocolate, including chocolate. ^1^ Letters indicate a significant (*p* < 0.01) difference between FS-ACCEPTABLE and FS-EXCESS based on GLM with survey design at three levels of adjustments: a, GLM adjusted for age, gender and energy intake; b, GLM adjusted for age, gender, energy intake, smoker status, BMI and socio-professional status; c, GLM adjusted for age, gender, energy intake, smoker status, BMI, socio-professional status, composition of the family and sitting time; ^2^ GLM to test differences in dietary intakes between observed and optimized diets, with survey design adjusted for age, gender, energy intake, smoker status, BMI, socio-professional status, composition of the family and sitting time.

## Supplementary Materials

The following are available online at http://www.mdpi.com/2072-6643/9/2/162/s1, Figure S1: Participant flow chart, Table S1: List of nutritional constraints included in the ID models, Table S2: Single nutrient ratios for MAR and MER for the total sample and for FS-ACCEPTABLE and FS-EXCESS groups (mean ± SD), Supplemental Methods: List of changes made to the previously published Individual Diet models.
