# Supplementary material for: Individual Diet Modeling Shows How to Balance the Diet of French Adults with or without Excessive Free Sugar Intakes
Source: Nutrients. 2017 Feb 20;9(2):162. doi: 10.3390/nu9020162 (PMC5331593; doi:10.3390/nu9020162)
Supplement: Supplementary file 1 [file nutrients-09-00162-s001.docx]

Supplementary Materials: Individual Diet Modeling Shows How to Balance the Diet of French Adults with or without Excessive Free Sugar Intakes

Anne Lluch, Matthieu Maillot, Rozenn Gazan, Florent Vieux, Fabien Delaere, Sarah Vaudaine and Nicole Darmon

1. Supplemental Methods: List of Changes Made to the Previously Published Individual Diet Models [1]

1.1. Nutritional Constraints

Sodium, free sugars and saturated fatty acids were limited to the maximal recommended values or to the observed intakes when they were lower than the maximal recommended values. A constraint imposing a minimal amount of water as a nutrient (H_2_O) was added and was set to the EFSA opinion adequate intake (at least 2000 g/day for women and at least 2500 g/day for men) [2].

1.2. Food Variables

The list of variables was extended to tea, coffee and drinking water. However, calorie-free drinks (<4 kcal/100 g) and all mineral water but not tap water were removed from the non-repertoire food variables to encourage the addition of tap water to reach the recommended intake of water as a nutrient (H_2_O).

1.3. Objective Function

To limit the increase in fortified foods and to reach the minimal requirement of H_2_O intake by selecting primarily tap water, positive deviations of fortified foods (i.e., ready-to-eat cereals) and hot drinks (tea, coffee and hot chocolate) were taken into account in the minimization. The weighting coefficients of non-repertoire foods were also modified to further penalize those foods consumed by fewer than 10% of individuals.

1.4. Total Weight Constraint

As previously described, the total weight of the modeled diet was limited to 115% of total diet weight of the observed diet [1]. However, in this upgraded version, calorie-free drinks were excluded from the calculation of total diet weight. This decision was made to avoid competition between calorie-free drinks and nutrient-dense foods with low energy content.


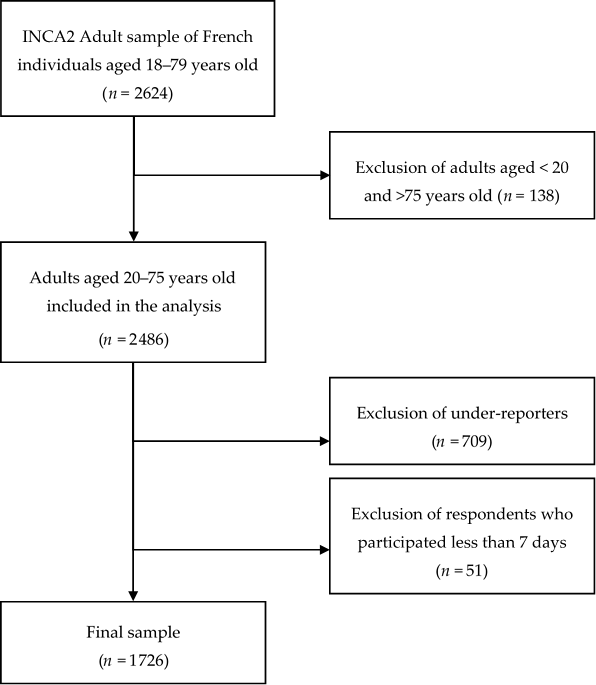


**Figure S1.** Participant flow chart.

**Table S1**. List of nutritional constraints included in the ID models ^1^.

| **Nutrients** | **Constraints** | **Reference** |
| --- | --- | --- |
| H_2_O, g/d | ≥2500 ^2^ | [2] |
| Proteins, g/kg/day | ≥0.83 | [3] |
| Lipids, % of total energy | 20–35 | [4] |
| Carbohydrates, % of total energy | 50–75 | [4] |
| Cholesterol, mg/day | ≤300 or OI ^3^ | [4] |
| *alpha*-Linolenic acid, % of total energy | ≥0.5 | [4] |
| Linoleic acid, % of total energy | 2.5–9 | [4] |
| DHA plus EPA, g/day | ≥0.25 | [4] |
| Omega-3 fatty acids, % of total energy | 0.5–2 | [4] |
| PUFAs, % of total energy | 6–11 | [4] |
| Saturated fatty acids, % of total energy | ≤10 or OI ^3^ | [4] |
| Free sugar, % of total energy | ≤10 or OI ^3^ | [5] |
| Sodium, mg/day | 1500–2759 ^4^ or OI ^3^ | [6] |
| Fiber, 10 vitamins, 9 minerals ^5^ | ≥EAR ^6^ or OI ^3^ or ANC ^6^ | [7] |

^1^ In all models, optimized diets were isoenergetic with observed diets, and the total weight was constrained between 85% and 115% of observed total weight; ^2^ Scientific opinion of the EFSA on the adequate intake of water as a nutrient, i.e. H_2_O: 2500 g was the minimal daily amount recommended for men. For women, this minimum amount was of 2000 g; ^3^ OI means observed intake; ^4^ Nordic nutrient recommendations: a total of 2759 mg (i.e., 7 g NaCl) was the upper limit for men. For women, the upper limit was 2365 mg (ie, 6 g NaCl); ^5^ The 10 vitamins were vitamin A, thiamine, riboﬂavin, niacin, panthotenic acid, vitamin B6, folates, vitamin B12, ascorbic acid, and vitamin E. The nine minerals were calcium, phosphorus, potassium, iron, magnesium, zinc, copper, iodine, and selenium; ^6^ EAR values = 77% of RDA values. Constraints took into account the gender and age of each individual, and the amount of the OI for each nutrient. The minimum levels imposed were as follows: at least the EAR when the OI was lower than the EAR, at least the RDA when the OI was greater than the RDA, and at least the OI when the amount was between the EAR and the RDA [1]. Values of the French RDA used in this study for the 10 vitamins and the nine minerals have been published [1].

**Table S2.** Single nutrient ratios for MAR and MER for the total sample and for FS-ACCEPTABLE and FS-EXCESS groups (mean ± SD).

|  | **ALL** | | **FS-ACCEPTABLE** | | **FS-EXCESS** | |  | | |
| --- | --- | --- | --- | --- | --- | --- | --- | --- | --- |
|  | **Mean** | ± **SD** | **Mean** | ± **SD** | **Mean** | ± **SD** | ***p* ^1^** | ***p* ^2^** | ***p* ^3^** |
| **Ratios used for the MAR** |  |  |  |  |  |  |  |  |  |
| Proteins | 1 | ±0.02 | 1 | ±0.02 | 1 | ±0.03 | 0.5728 | 0.6705 | 0.6637 |
| *alpha*-Linolenic acid | 0.61 | ±0.21 | 0.62 | ±0.21 | 0.59 | ±0.20 | 0.0092 | 0.0125 | 0.0153 |
| Linoleic acid | 0.94 | ±0.12 | 0.94 | ±0.11 | 0.94 | ±0.13 | 0.035 | 0.0447 | 0.0383 |
| DHA | 0.76 | ±0.31 | 0.80 | ±0.29 | 0.69 | ±0.33 | 0.0001 | <0.0001 | <0.0001 |
| Fiber | 0.62 | ±0.18 | 0.65 | ±0.19 | 0.58 | ±0.17 | <0.0001 | <0.0001 | <0.0001 |
| Vitamin A | 0.95 | ±0.13 | 0.96 | ±0.12 | 0.93 | ±0.14 | <0.0001 | <0.0001 | <0.0001 |
| Thiamin | 0.89 | ±0.14 | 0.90 | ±0.13 | 0.88 | ±0.14 | <0.0001 | <0.0001 | <0.0001 |
| Riboflavin | 0.92 | ±0.13 | 0.92 | ±0.12 | 0.91 | ±0.13 | 0.1774 | 0.2422 | 0.2200 |
| Niacin | 0.99 | ±0.06 | 0.99 | ±0.05 | 0.98 | ±0.07 | 0.0121 | 0.0274 | 0.0234 |
| Vitamin B6 | 0.91 | ±0.13 | 0.92 | ±0.12 | 0.88 | ±0.14 | <0.0001 | <0.0001 | <0.0001 |
| Folates | 0.84 | ±0.17 | 0.86 | ±0.16 | 0.81 | ±0.18 | <0.0001 | <0.0001 | <0.0001 |
| Vitamin B12 | 0.99 | ±0.04 | 0.99 | ±0.04 | 0.99 | ±0.04 | 0.3299 | 0.3989 | 0.4576 |
| Acid ascorbic | 0.72 | ±0.26 | 0.72 | ±0.25 | 0.72 | ±0.28 | 0.0377 | 0.0186 | 0.0424 |
| Vitamin E | 0.86 | ±0.18 | 0.87 | ±0.18 | 0.84 | ±0.19 | 0.0269 | 0.0387 | 0.0187 |
| Vitamin D | 0.52 | ±0.23 | 0.54 | ±0.24 | 0.49 | ±0.22 | 0.0001 | 0.0002 | 0.0002 |
| Calcium | 0.83 | ±0.19 | 0.81 | ±0.20 | 0.85 | ±0.17 | 0.7482 | 0.8717 | 0.9682 |
| Potassium | 0.88 | ±0.14 | 0.89 | ±0.14 | 0.87 | ±0.14 | 0.001 | 0.0028 | 0.0032 |
| Magnesium | 0.78 | ±0.16 | 0.78 | ±0.16 | 0.78 | ±0.16 | 0.2254 | 0.3535 | 0.2919 |
| Iodine | 0.80 | ±0.18 | 0.81 | ±0.18 | 0.79 | ±0.19 | 0.0062 | 0.0121 | 0.0079 |
| Selenium | 0.98 | ±0.07 | 0.98 | ±0.07 | 0.97 | ±0.07 | 0.102 | 0.1389 | 0.1058 |
| Copper | 0.82 | ±0.17 | 0.83 | ±0.16 | 0.81 | ±0.17 | 0.1004 | 0.1175 | 0.0823 |
| Zinc | 0.87 | ±0.15 | 0.88 | ±0.14 | 0.86 | ±0.15 | <0.0001 | 0.0001 | 0.0002 |
| Iron | 0.81 | ±0.21 | 0.82 | ±0.21 | 0.80 | ±0.22 | 0.2762 | 0.4338 | 0.4759 |
| **Ratios used for the MER** |  |  |  |  |  |  |  |  |  |
| Saturated fatty acids | 1.6 | ±0.5 | 1.59 | ±0.52 | 1.62 | ±0.48 | 0.0081 | 0.0089 | 0.0053 |
| Free sugars | 1.26 | ±0.5 | 1.03 | ±0.1 | 1.59 | ±0.64 | <0.0001 | <0.0001 | <0.0001 |
| Sodium | 1.11 | ±0.2 | 1.13 | ±0.22 | 1.07 | ±0.15 | <0.0001 | <0.0001 | <0.0001 |

^1^ GLM adjusted for age, gender and energy intake; ^2^ GLM adjusted for age, gender, energy intake, smoker status, BMI and socio-professional status; ^3^ GLM adjusted for age, gender, energy intake, smoker status, BMI, socio-professional status, composition of the family and sitting time.

References

1. Maillot, M.; Vieux, F.; Amiot, M.J.; Darmon, N. Individual diet modeling translates nutrient recommendations into realistic and individual-specific food choices. *Am. J. Clin. Nutr.* 2010, 91, 421–430.
2. European Food Safety Authority. Panel on dietetic products and allergies. *EFSA J.* 2010, 8, 1459, doi: 10.2903/j.efsa.2010.1459.
3. World Health Organization. *Protein and Amino Acid Requirements in Human Nutrition*; Report of a Joint FAO/WHO/UNU Expert Consultation (WHO Technical Report Series 935); World Health Organization: Geneva, Switzerland, 2007; pp. 1–265.
4. World Health Organization (Geneva). *Diet, Nutrition and the Prevention of Chronic Diseases*; Report of the Joint WHO/FAO Expert Consultation (WHO Technical Report Series 916); World Health Organization: Geneva, Switzerland, 2002.
5. World Health Organization (Geneva). Guideline: Sugars intake for adults and children. World Health Organization: Geneva, Switzerland, 2015. Available online: http://apps.who.int/iris/bitstream/10665/149782/1/9789241549028_eng.pdf (accessed on 17 February 2017).
6. Becker, W.; Lyhne, N.; Pedersen, A.; Aro, A.; Fogelholm, M.; Þhórsdottir, I.; Alexander, J.; Anderssen, S.; Meltzer, H.; Pedersen, J. Nordic Nutrition Recommendations 2004—integrating nutrition and physical activity. *Scand. J Nutr.* **2004**, *48*, 178–187.
7. Martin, A. *Apports nutritionnels conseillés pour la population Française*, 3rd ed.; (Recommended dietary intakes for the French population 3rd edition);TEC&DOC.: Paris, France, 2001.
